# Prediction of the 1-Year Risk of Incident Lung Cancer: Prospective Study Using Electronic Health Records from the State of Maine

**DOI:** 10.2196/13260

**Published:** 2019-05-16

**Authors:** Xiaofang Wang, Yan Zhang, Shiying Hao, Le Zheng, Jiayu Liao, Chengyin Ye, Minjie Xia, Oliver Wang, Modi Liu, Ching Ho Weng, Son Q Duong, Bo Jin, Shaun T Alfreds, Frank Stearns, Laura Kanov, Karl G Sylvester, Eric Widen, Doff B McElhinney, Xuefeng B Ling

**Affiliations:** 1 Shandong Provincial Key Laboratory of Network Based Intelligent Computing University of Jinan Jinan China; 2 Department of Surgery Stanford University Stanford, CA United States; 3 Department of Oncology The First Hospital of Shijiazhuang Shijiazhuang China; 4 Department of Cardiothoracic Surgery Stanford University Stanford, CA United States; 5 Clinical and Translational Research Program Betty Irene Moore Children's Heart Center Lucile Packard Children’s Hospital Palo Alto, CA United States; 6 Department of Bioengineering University of California Riverside, CA United States; 7 West China-California Multiomics Research Center West China Hospital Sichuan University Chengdu China; 8 Department of Health Management Hangzhou Normal University Hangzhou China; 9 Healthcare Business Intelligence Solutions Inc Palo Alto, CA United States; 10 Lucile Packard Children’s Hospital Palo Alto, CA United States; 11 HealthInfoNet Portland, ME United States

**Keywords:** lung cancer, risk prediction model, electronic health records, prospective study

## Abstract

**Background:**

Lung cancer is the leading cause of cancer death worldwide. Early detection of individuals at risk of lung cancer is critical to reduce the mortality rate.

**Objective:**

The aim of this study was to develop and validate a prospective risk prediction model to identify patients at risk of new incident lung cancer within the next 1 year in the general population.

**Methods:**

Data from individual patient electronic health records (EHRs) were extracted from the Maine Health Information Exchange network. The study population consisted of patients with at least one EHR between April 1, 2016, and March 31, 2018, who had no history of lung cancer. A retrospective cohort (N=873,598) and a prospective cohort (N=836,659) were formed for model construction and validation. An Extreme Gradient Boosting (XGBoost) algorithm was adopted to build the model. It assigned a score to each individual to quantify the probability of a new incident lung cancer diagnosis from October 1, 2016, to September 31, 2017. The model was trained with the clinical profile in the retrospective cohort from the preceding 6 months and validated with the prospective cohort to predict the risk of incident lung cancer from April 1, 2017, to March 31, 2018.

**Results:**

The model had an area under the curve (AUC) of 0.881 (95% CI 0.873-0.889) in the prospective cohort. Two thresholds of 0.0045 and 0.01 were applied to the predictive scores to stratify the population into low-, medium-, and high-risk categories. The incidence of lung cancer in the high-risk category (579/53,922, 1.07%) was 7.7 times higher than that in the overall cohort (1167/836,659, 0.14%). Age, a history of pulmonary diseases and other chronic diseases, medications for mental disorders, and social disparities were found to be associated with new incident lung cancer.

**Conclusions:**

We retrospectively developed and prospectively validated an accurate risk prediction model of new incident lung cancer occurring in the next 1 year. Through statistical learning from the statewide EHR data in the preceding 6 months, our model was able to identify statewide high-risk patients, which will benefit the population health through establishment of preventive interventions or more intensive surveillance.

## Introduction

### Background

Lung cancer is the most common cancer and leading cause of cancer death worldwide [1,2]. In 2018, the number of new cases of lung and bronchus cancer was estimated to be 234,030 (13.5% of all new cancer cases); an estimated 154,050 people will die of this disease (25.3% of all cancer-related deaths) in the United States alone [3]. Statistics on survival in people with lung cancer vary depending on the stage of the cancer when it is diagnosed. Early captures at stage I have a 56.3% 5-year survival rate, which decreases to 4.7% by stage III in the United States, based on data from the Surveillance, Epidemiology, and End Results Program 18, 2008-2014 [3]. Most people with lung cancer are diagnosed at a late stage when curative treatment is less effective. Therefore, early detection and timely disease intervention play an important role in reducing the mortality rate of lung cancer.

Annual low-dose computed tomography (LDCT) screening is a viable screening tool for early lung cancer detection. The US-based National Lung Screening Trial demonstrated that LDCT screening reduced lung cancer mortality by 20% relative to conventional chest x-ray screening [4]. However, the screening criteria for LDCT are only age (55-74 years) and smoking history (>30 pack-years, <15 years quit time) [5]. Therefore, a lot of patients take unnecessary tests, which is a serious misuse of social resources; at the same time, many people who seem healthy have been missed [6]. However, so far, there is no tool aimed at the whole population. An effective risk prediction model is critically needed for the initial screening of high-risk patients at the population level, which would hold promise for seeking out those high-risk individuals for further LDCT examination, ensuring that resources are focused on those who are most likely to benefit from them.

Accurate lung cancer risk prediction models would facilitate early diagnoses, decrease mortality rates, and reduce overall costs, ultimately benefiting patients, clinicians, and health care providers.

### Possible Limitations of Existing Lung Cancer Risk Prediction Models

Many lung cancer risk prediction models have been proposed [7-21]; however, the clinical needs have not been sufficiently addressed [22]. Most of the recent lung cancer risk prediction models were developed (1) with a small number of risk predictors (N<15) [7,9-21], (2) with a small sample size and using data from only a single medical facility [12,17,21], and (3) with a focus on a particular subgroup of the population (eg, age>45 or smokers) [7,9-14,16,18-20] and the lack of generalizability across the heterogenous population [7-20]. Furthermore, most prior studies used smoking status (eg, smoking duration, smoking intensity, and years since cessation) as a risk factor and predictor [10,12,15,17,20,21], which may not be readily available in many of the medical data sources.

The use of electronic health records (EHRs) has increased dramatically in recent years. The large size and high-dimensional clinical patient information captured in EHRs may be more reflective of the characteristics of the general population than those of cohort studies based on a targeted subgroup of limited profiles. EHRs provide a unique opportunity to understand the health care status at the population level [23]. EHR-based models were developed for diseases including but not limited to type 2 diabetes, chronic kidney disease, and hypertension [23-32]; however, no model was reported to predict new incident lung cancer based on statewide EHR data in the United States.

### Aim

Our study aimed to prospectively estimate the future 1-year risk of new incident lung cancer in a US state population. The predictive model uses the preceding 6 months’ EHR information including current health conditions, diagnosed diseases, symptoms, laboratory tests, medication history, clinical utilization measures, and social determinants. The model outputs a risk score that describes the probability of a diagnosis of new incident lung cancer in the next 1 year. The risk scores stratify patients into low-, medium-, and high-risk categories, by which limited health care resources can be targeted to high-risk groups to allow proactive intervention, which can ultimately allow early detection of cancer and reduction of regional/statewide lung cancer mortality rates.

## Methods

**Figure 1:**
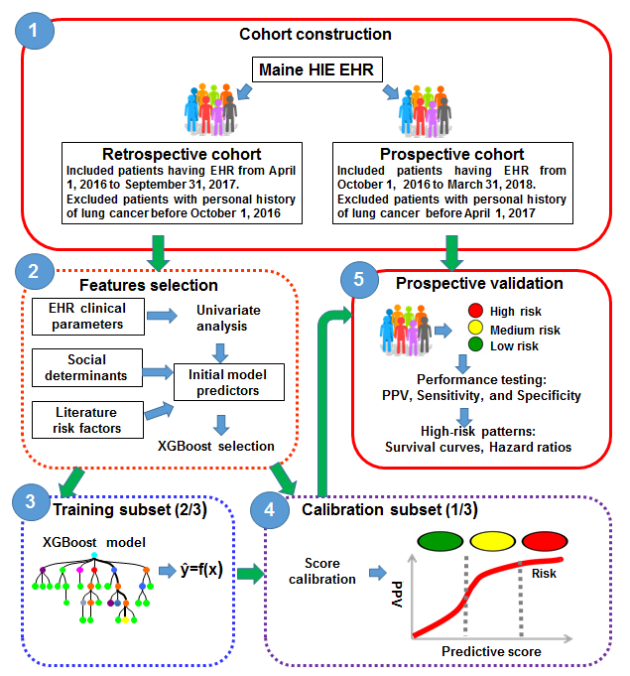
A workflow to develop the new incident lung cancer risk prediction model. EHR: electronic health record; HIE: health information exchange; PPV: positive predictive value. XGBoost: Extreme Gradient Boosting.

A workflow to develop the new incident lung cancer risk prediction model is provided in Figure 1. This study includes five steps from cohort construction to prospective validation.

### Ethics Statement

Protected personal health information was removed before the process of analysis and publication. Because this study analyzed deidentified data, it was exempted from ethics review by the Stanford University Institutional Review Board (March 20, 2017).

### Population and Data Sources

The EHRs of Maine Health Information Exchange (HIE; HealthInfoNet) dataset cover records of nearly 95% of the population of the state of Maine [33]. The study included patients who visited any care facility, 35 hospitals, 34 federally qualified health centers, and more than 400 ambulatory practices in the Maine state, from April 1, 2016, to March 31, 2018.

### Lung Cancer Definition

Lung cancer in this study was defined using International Classification of Diseases, 10th Revision, Clinical Modification (ICD-10-CM) diagnosis codes. The diagnosis codes included category C34 (malignant neoplasm of bronchus and lung), C39 (malignant neoplasm of other and ill-defined sites in the respiratory system and intrathoracic organs), and C46.5 (Kaposi sarcoma of the lung).

### Cohort Construction

This study contains a retrospective cohort and a prospective cohort (Figure 1). The retrospective cohort contained 873,598 patients with EHRs from April 1, 2016, to September 31, 2016, and 1091 of them developed lung cancer in the next year (from October 1, 2016, to September 31, 2017). Patients were excluded from the retrospective cohort if there was any record of a lung cancer diagnosis before October 1, 2016. The prospective cohort included 836,659 patients from October 1, 2016, to March 31, 2017, and 1167 of them were diagnosed with lung cancer in the next year (from April 1, 2017, to March 31, 2018). Patients with a history of lung cancer before April 1, 2017, were excluded from the prospective cohort.

### Feature Selection

The clinical parameters extracted from EHRs included demographic information, disease diagnoses (primary and secondary), symptoms and procedures (coded using ICD-10-CM), laboratory test results (coded by Logical Observation Identifier Names and Codes and labeled as abnormal or normal according to thresholds provided by each facility participating in the HIE network), clinical utility records, and outpatient medication prescriptions (coded according to the National Drug Code and referred to the number of prescriptions for a particular medicine during the past 6 months). We also extracted a number of accessible social determinants from the US census website [34] using zip code or county name in the Advanced Search of American FactFinder (Multimedia Appendix 1). These social determinants were mapped to the EHR database through a patient’s zip code. In addition, features associated with lung cancer identified by previous studies were also extracted as risk factors. Those risk factors included demographics (ie, age and gender), smoking, pulmonary diseases (ie, chronic obstructive pulmonary disease [COPD], chronic bronchitis, emphysema, and pneumonia), symptoms (ie, hemoptysis, cough, and chest pain), and abnormal laboratory test results (ie, C-reactive protein and fibrinogen). Overall, there were 33,788 features in our original data pool.

Given that high-dimensional EHR data are sparse and subject to noisy and missing data, a feature selection process was adopted before model construction. The process had included univariate analysis and XGBoost selection. For EHR clinical parameters, a univariate correlation filtering analysis was adopted to remove features that are not significantly related to lung cancer (*P*>.05). Specifically, the Cochran-Mantel-Haenszel test [35], capable of testing the association between a binary predictor and a binary outcome while taking into account the stratification, was applied to investigate the association between the binary features and the targeted outcome under age-group strata. The Cochran-Armitage trend test [35,36], also called the Chi-square test for trend, was used in the analysis of categorical data to assess the strength of the association between an ordinal variable with *k* categories. Univariate logistic regression [37] was used to assess features that are continuous variables. Social determinants, literature risk factors, and features identified by our univariate analysis were combined (N=346) for the downstream XGBoost analytics. XGBoost adopted the approximate greedy algorithm to split trees by sorting and picking features on each node to optimize purity at each splitting level. The algorithm can output estimates of feature importance after going through the training process [38].

### Model Construction

Samples in the retrospective cohort (873,598 patients) were randomly split into 2 subsets for training (582,398 patients) and calibration (291,200 patients) purposes. The model construction, using the retrospective dataset, was accomplished in two phases: (1) the training subset was used to develop the initial model and generate predictive estimates and (2) the calibration subset was used to convert predictive estimates to risk scores for each patient.

#### Training

XGBoost [38], a gradient tree boosting algorithm, was applied to develop a prediction model. XGBoost algorithm is designed to discover statistical patterns in high-dimensional and multivariate datasets and is able to handle nonlinear correlations and random errors both in input features and the output variable [39]. We used binary classification with logistic objective function for the predictive estimate. The output predictions were probability confidence scores in (0,1), corresponding to the probability of receiving a new diagnosis of incident lung cancer within the next 1 year. The objective was implemented in the “xgboost” package for the R language provided by the creators of the algorithm. The output of the algorithm can be written as 
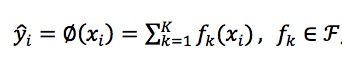
 where *F* represents the space of a set of classification trees and *K* is the maximum number of trees (*K*=500 in this study). Each f_k_ corresponds to an independent tree, and the maximum depth of each tree was set to 5 in this study. The final prediction was calculated by summing up the scores of all the individual trees. To avoid overfitting, the model at the *t*-th iteration was trained to minimize the following item, 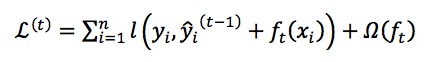 where 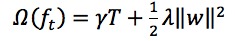 was the prediction of the *i*-th instance at the *t-1*-th iteration, and *l* is a differentiable convex loss function. The term Ω indicates the penalty of the model complexity and is defined as 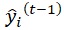 where γ and are parameters controlling penalty for the number of leaves *T* and magnitude of leaf weights *w,* respectively. The penalty parameter is selected by cross-validation from the values ranging from λ*=10e*^2^ to λ*=10e*^-2^, essentially covering the full range of scenarios from the null model containing no penalty to the least squares fit.

An approximate algorithm was used to split the finding. It first proposes candidate splitting points according to percentiles of feature distribution, following which splitting points were chosen to optimize purity at the next level.

#### Calibration

A calibration process was launched to map the predictive estimates of XGBoost to a measure of positive predictive values (PPVs) [40] in the retrospective cohort. It provided a universal, standardized risk measure. A PPV of a corresponding predictive estimate 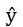 was defined as the proportion of new incident lung cancer events in the cohort with predictive estimates the same as or larger than 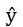. Thus, PPVs could be interpreted as risk scores. Following that, we further ranked individuals by their risk scores from low to high, and two risk thresholds were applied to subgroup all patients into low-risk, medium-risk, and high-risk groups.

The scores after calibration were converted to relative risks. The relative risk of each individual was calculated by dividing the score of the individual by the mean score of all patients in the cohort. The relative risk measured the ratio of the probability of having lung cancer to the population baseline. The higher the relative risk, the higher was the probability of receiving a diagnosis of lung cancer in the next year.

### Prospective Validation

The model was tested with the prospective cohort (836,659 patients). Performance of the model was investigated within each risk category in terms of PPV, sensitivity, and specificity. The receiver operating characteristic (ROC) curve and the area under the curve (AUC) were also calculated. Relative risk of a subgroup (the ratio of the mean score of the patients in the subgroup to the population mean) was used to measure the increase or decrease in the chance of obtaining a new diagnosis of lung cancer in the next year for patients in the subgroup, compared to the population baseline.

Age- and gender-adjusted odds ratios (ORs) between cases and controls were calculated for top features using logistic regression. Clinical patterns stratified by risk categories were explored and compared. Multivariable Cox regression was used for subpopulation comparison. Spearman rank correlations were performed to assess the correlation between social determinants and the next 1-year risk of lung cancer.

## Results

### Baseline Characteristics

Baseline demographic and clinical features of the retrospective and prospective cohorts are summarized in Table 1. Most characteristics were similarly distributed between these two cohorts.

### Model Performance

**Figure 2:**
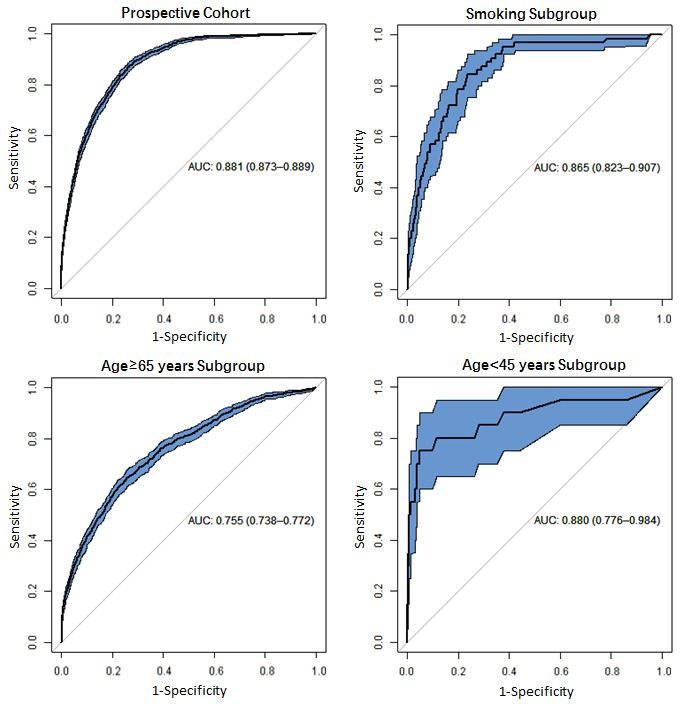
The receiver operating characteristic curves derived from the prospective cohort, smoking subgroup, age≥65 years subgroup, and age<45 years subgroup. The 95% CI of each receiver operating characteristic curve is indicated by the blue shaded area and the AUC (with 95% CI) of each subgroup is listed under each receiver operating characteristic curve. AUC: area under the curve.

By applying the XGBoost algorithm on the EHR-based data, the prediction model reached an AUC of 0.881 (95% CI 0.873-0.889) in the prospective cohort (Figure 2). The model also had effective discriminatory power within patient subgroups: (1) the smoking subgroup (14,248/836,659, 1.7%) with an AUC of 0.865 (95% CI 0.823-0.907), (2) subgroup of age≥65 years (220,702/836,659, 26.4%) with an AUC of 0.755 (95% CI 0.738-0.772), and (3) subgroup of age<45 years (366,752/836,659, 43.8%) with an AUC of 0.880 (95% CI 0.776-0.984; Figure 2). Predictive scores of cases and controls in the prospective cohort were analyzed using the Wilcoxon test [41] (*P*<.001), supporting the statistical importance of our results.

To explore the effectiveness and advantages of our model, we compared the predictive performance of our XGBoost algorithm with a few state-of-the-art existing predictive algorithms in the prospective cohort. Algorithms included RandomForest [42], Boosting [43], Support Vector Machine [44], Lasso [45], and k-nearest neighbors (KNN) [46]. Multimedia Appendix 2 compared the ROC AUCs with the 95% CIs of our model and other existing predictive algorithms to predict the future risk of new incident lung cancer in the next 1 year. Algorithm performances (ROC AUC) were compared and the differences were quantified using the deLong method [47]. For all comparisons, our model outperformed other models, with significantly superior predictive performance (*P*<.001). We also compared our model’s predictive performance to other feature selection methods including information gain and Gini index methods. The results were shown in Multimedia Appendix 3. Our comparative results showed that the model predictive performance based on our feature selection method outperformed the performance based on the other methods in terms of the ROC AUC (*P*<.001; *P* values calculated by the deLong method to compare the AUCs).

**Table 1:** Baseline characteristics of the retrospective cohort (N=873,598) and prospective cohort (N=836,659).

Characteristic		Retrospective cohort, n (%)	Prospective cohort, n (%)
**Age (years)**			
	<45	385,009 (44.1)	366,752 (43.8)
	45-54	116,655 (13.4)	109,986 (13.1)
	55-64	143,960 (16.5)	139,219 (16.6)
	≥65	227,974 (26.1)	220,702 (26.4)
**Gender**			
	Male	386,251 (44.2)	369,022 (44.1)
	Female	487,347 (55.8)	467,637 (55.9)
Smoking^a^		16,611 (1.9)	14,248 (1.7)
Other cancer history		59,239 (6.8)	72,039 (8.6)
**Pulmonary disease**			
	COPD^b^	32,180 (3.7)	36,221 (4.3)
	Pneumonia	9,896 (1.1)	12,179 (1.5)
	Other respiratory disorders^c^	5131 (0.6)	5738 (0.7)
**Other chronic disease**			
	Diabetes	73,854 (8.5)	70,005 (8.4)
	CVDs^d^	166,088 (19)	161,685 (19.3)
	CKD^e^	18,458 (2.1)	18,912 (2.3)
**Symptom**			
	Cough	26,574 (3)	36,810 (4.4)
	Chest pain	32,101 (3.7)	35,057 (4.2)
	Hemoptysis	770 (0.1)	981 (0.1)
	Dyspnea	4071 (0.5)	3755 (0.5)
	Pleural effusion	2024 (0.2)	2356 (0.3)
	Abnormal weight loss	6136 (0.7)	5801 (0.7)
**Abnormal laboratory test**			
	C-reactive protein test	11,613 (1.3)	8,517 (1)
	Leukocytes count	90,131 (10.3)	71,694 (8.6)
	Platelets	69,334 (7.9)	51,477 (6.2)
	Glomerular filtration rate	21,446 (2.5)	18,695 (2.2)
	Glucose in serum or plasma	137,575 (15.8)	103,671 (12.4)

^a^Smoking was defined with a diagnosis code of Z72_2 from the International Classification of Diseases, 10th Revision, Clinical Modification.

^b^COPD: chronic obstructive pulmonary disease (including chronic bronchitis and emphysema).

^c^Other respiratory disorders were defined with a diagnosis code of J98 from the from the International Classification of Diseases, 10th Revision, Clinical Modification.

^d^CVD: cardiovascular disease (including hypertension, coronary artery disease, peripheral vascular disease, arrhythmia, and abdominal aortic aneurysm).

^e^CKD: chronic kidney disease.

**Figure 3:**
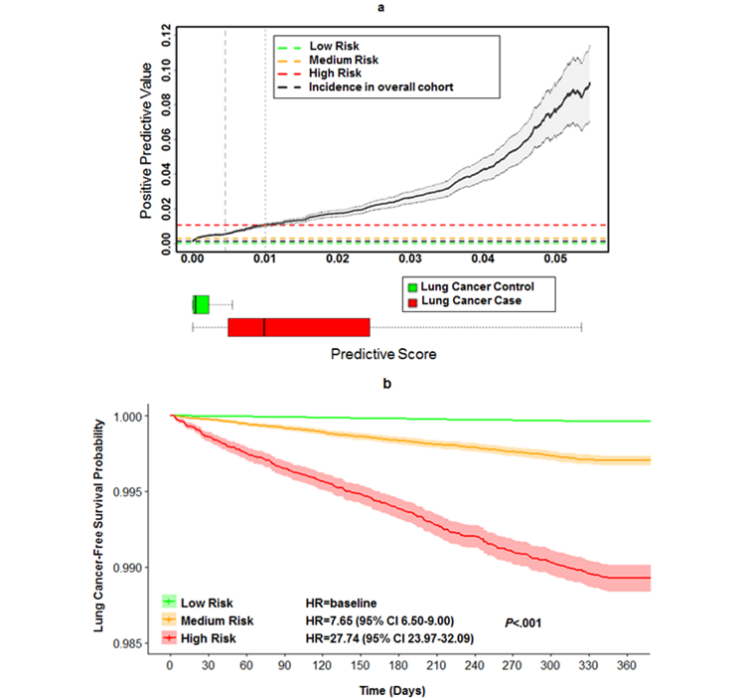
(a) Stratification of patients in the prospective cohort. Positive predictive value was plotted as a function of the predictive score. Two thresholds of 0.0045 and 0.01 were applied to stratify the population into low-, medium- and high-risk categories. (b) Survival curves of the three risk categories. HR: Hazard Ratio.

The relationship between the PPVs and predictive scores with the prospective cohort is shown in Figure 3 a. Vertical dashed lines indicate two thresholds of 0.0045 and 0.01 of the predictive scores to group all patients into three risk categories (low, medium, and high). Horizontal dashed lines indicate the incidence of lung cancer in the overall cohort (0.14%, black), low-risk category (0.04%, green), medium-risk category (0.3%, orange), and high-risk category (1.07%, red). The 95% CI of the PPV curve is indicated by the gray shaded area. The box plots at the bottom show the distributions of predictive scores. The performance of our model within each risk category in terms of PPV, sensitivity, specificity, and mean relative risk is shown in Multimedia Appendix 4. Among the 1167 patients in the prospective cohort with confirmed lung cancer in the next 1 year, about half (579/1167, 49.61%) were correctly classified into the high-risk category (with a score≥0.01), and only 22.45% (262/1167) of them were classified into the low-risk category (with a score<0.0045). The relative risk showed a monotonic increase from the low-risk category (0.28) to the high-risk category (7.7). We also calculated the sensitivity and specificity of our model based on the best cut-off threshold for predictive scores, which was defined as the point at which the Youden index (sensitivity+specificity-1) [48] is maximum. After considering the Youden index, the best cut-off point was 0.0029, and the corresponding sensitivity and specificity of our model were 0.8363 and 0.7681, respectively.

A survival analysis using univariable cox regression was performed on each risk category to further evaluate the model performance. Three distinct survival curves stratified patients in terms of lung cancer hazard (*P*<.001), yielding a hazard ratio (HR) as high as 27.74 (95% CI 23.97-32.09) for the high-risk category relative to the low-risk group (Figure 3 b). In addition, our model identified 41.82% (289/691) of high-risk patients 6 months or more prior to assignment of a lung cancer diagnosis code. A total of 68.02% (470/691) of lung cancer cases were identified as high risk at least 3 months before the confirmatory diagnosis was made by physicians.

From the original 33,788 features, 346 features survived from the first step of feature selection process (filtered by univariate analysis) and 118 features were identified by XGBoost algorithm as final predictors of the model (filtered by nonzero weight in algorithm). They consisted of two demographic features, 11 social determinations, 19 diagnostic diseases, 9 clinical symptoms, 28 laboratory tests, 37 medication prescriptions, and 12 clinical utilization measures. The top 60 features with their age-gender adjusted ORs or coefficients are listed in Multimedia Appendix 5. COPD, pneumonia, and other respiratory disorders were recognized as the pulmonary diseases most associated with lung cancer, with ORs of 4.978, 2.790, and 5.484, respectively. Other cancer history, cardiovascular diseases (CVDs), diabetes, and CKD were considered to be chronic diseases most associated with lung cancer, with ORs of 1.899, 1.329, 1.374, and 1.270, respectively. Smoking also had a strong association with lung cancer (OR=4.084). Hemoptysis, pleural effusion, cough, and abnormal weight loss were recognized as symptoms most associated with lung cancer, with ORs of 5.080, 4.130, 2.108, and 2.010, respectively. For abnormal laboratory test results, inflammation marker C-reactive protein was most associated with lung cancer (OR=1.771). Medications for treatment of chronic diseases and mental disorders, clinical utilization, and social determinants were also detected by the model as powerful predictors of incident lung cancer within the next year.

### Clinical Patterns Stratified by Risk Categories

Distribution patterns of impactful risk predictors were explored and compared among different risk categories: low risk (score 0-0.0045; 673,075 patients), medium risk (score 0.0045-0.01; 109,662 patients), and high risk (score 0.01-1; 53,922).

#### Age

In our study, age was aggregated into four distinct age groups (<45, 45-54, 55-64, and ≥65 years). A significant difference was found in the age distribution between the low- and the high-risk categories (Multimedia Appendix 7). In the low-risk category, younger individuals (<45) accounted for 54.16% (364,545/673,075), whereas older individuals (≥65 years) accounted for only 13.64% (91,815/673,075). In the high-risk category, the group aged ≥65 years constituted the largest subset (83.32%, 44,927/53,922), whereas the group aged <45 years constituted only 0.79% (426/53,922; Multimedia Appendix 6).

#### Diagnosed Diseases

History of pulmonary diseases and other chronic diseases also differed between low- and high-risk patients: 50.48% (27,219/53,922), 24.29% (13,100/53,922), and 21.62% (11,659/53,922) of the high-risk patients had CVDs, diabetes, and COPD, respectively, while 12.30% (82,757/673,075), 5.04% (33,927/673,075), and 1.98% (13,322/673,075) of the low-risk patients had these diseases, respectively (Multimedia Appendix 6).

**Figure 4:**
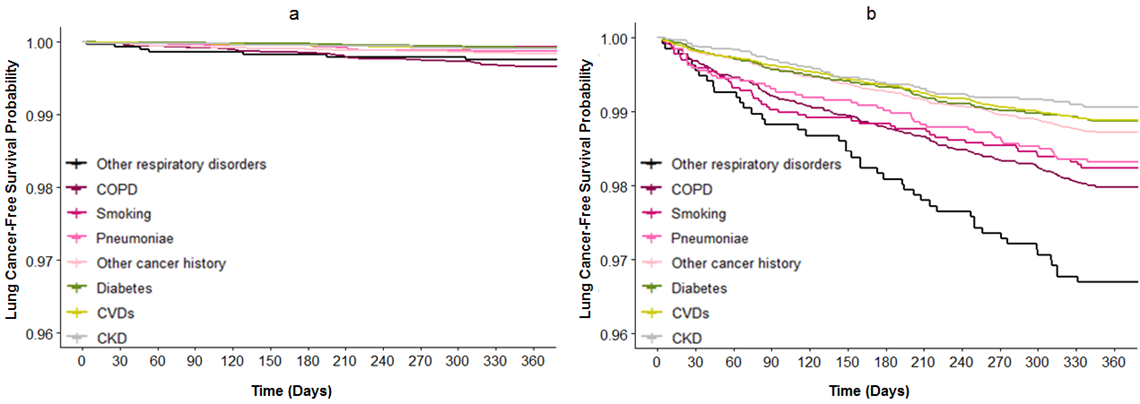
Time-to-diagnosis curves of the disease subgroup, smoking subgroup, and other cancer history subgroup for the low-risk (a) and high-risk (b) categories of the prospective cohort. Disease subgroups comprised patients who received diagnoses of COPD, pneumonia, other respiratory disorders, diabetes, CVDs, or CKD. CKD: chronic kidney disease; COPD: chronic obstructive pulmonary disease; CVD: cardiovascular disease.

The time-to-diagnosis curves were created using univariable Cox regression to explore lung cancer diagnoses in the high- and low-risk categories in different disease subgroups (ie, COPD, pneumonia, other respiratory disorders, CVDs, diabetes, and CKD), smoking subgroups, and other cancer history subgroups (Figure 4). In the high-risk category, 1.61% (47/2677) of patients with a smoking history and 1.28% (185/14477) of patients with other cancer history received diagnoses of lung cancer in the next 1 year. In addition, 1.68% (46/2734), 2% (235/11,659), and 3.3% (45/1363) of patients with pneumonia, COPD, other respiratory disorders, respectively, received diagnoses of lung cancer in the next 1 year. This probability remained around 1% for patients with CVDs, diabetes, and CKD. These results implied that patients with pulmonary diseases (ie, COPD, pneumonia, or other respiratory disorders) have higher risks for developing lung cancer than patients with other chronic diseases (ie., CVDs, diabetes, and CKD). In the low-risk category, more than 99.6% of the patients were free from development of lung cancer in the next 1 year, and the survival curve dropped faster for patients with pulmonary diseases than for those with other chronic diseases.

We also investigated the time-to-diagnosis curves for patients who only had pulmonary diseases and patients who had pulmonary diseases together with at least one other chronic disease (including diabetes, CVDs, CKD, and other cancer history). Results showed that a history of pulmonary disease together with other chronic diseases increased the risk of incident lung cancer (HR=1.7; Multimedia Appendix 8). In addition, 22.05% (11,890/53,922) of the high-risk patients had pulmonary diseases together with other chronic diseases, while 5.76% (3082/53,922) of the patients had pulmonary diseases only. In the low-risk category, these values were 1.45% (9732/673,075) and 1.95% (13,100/673,075), respectively.

#### Symptoms and Abnormal Laboratory Test Results

The model recognized 6 symptoms and 10 abnormal laboratory test results as powerful predictors of the 1-year lung cancer risk (Multimedia Appendix 5). Most were acute symptoms related to the respiratory system, and most of the laboratory test results were markers associated with inflammation and chronic diseases. The percentage of patients with these symptoms and abnormal laboratory test results gradually increased from the low- to high-risk category (Multimedia Appendix 6). Furthermore, 23.73% (12,798/53,922) and 39.62% (21,364/53,922) of people in the high-risk category had at least one symptom or one abnormal laboratory test result compared with 9.12% (61,368/673,075) and 11.67% (78,564/673,075) in the low-risk category, respectively.

#### Mental Disorders

The model identified 15 medications prescribed for mental disorders (eg, depression and anxiety disorders) as impactful features (Multimedia Appendix 5). Mental disease information was unavailable in HIE due to privacy concerns. Our study used mental disorder-related medications to explore the association between mental disorders and lung cancer risk. People with a history of mental disorder-related medications were significantly enriched in the high-risk group (Multimedia Appendix 6), with a total of 22.97% (12,388/53,922) in the high-risk category and 7.36% (49,537/673,073) in the low-risk category.

Survival analysis was performed according to mental health status (mental disorder or no mental disorder) in the subgroup that comprised people with at least one chronic disease diagnosis and the subgroup of patients with no history of chronic diseases (Multimedia Appendix 9). Presence of a mental disorder was associated with an increased risk of lung cancer in both subgroups before and after adjustment for age, gender, and smoking (*P*<.001). Therefore, the presence of mental disorders increased the risk of lung cancer independent of chronic diseases, age, gender, and smoking factors.

#### Clinical Utilization Indicators

We compared the clinical utilization indicators in our study across three risk categories. These utilization indicators of patients such as outpatient visits, emergency visits, inpatient admissions, inpatient days, clinical cost, and number of chronic diseases in the past 6 months gradually increased from the low- to high-risk categories (Multimedia Appendix 6).

We further compared patients in the high- and low-risk categories by the average clinical costs in the past 6 months in 8 disease subgroups (including COPD, CVDs, pneumonia, other respiratory disorders, diabetes, CKD, mental disorders, and other cancer history) stratified by the average number of chronic diseases (Multimedia Appendix 10). The circles in Multimedia Appendix 10 were formed by 8 disease subgroups under the low-risk (green circle) and high-risk (red circle) categories. The circle size indicates the proportion of the disease subgroup under each risk category. Reference groups consisted of patients with no diagnosis of any of these chronic diseases. It is obvious that the low-risk patients and high-risk patients were separated by the number of the chronic diseases. High-risk patients, in general, had a higher chronic disease burden and thus higher clinical costs.

#### Social Determinants

In our study, the social determinants were derived from zip code or county-based census and were recognized as community-level social and environmental indicators. Spearman rank correlations were used to investigate the association between social determinants and lung cancer risk (Multimedia Appendix 11). Parameters related to a decreased risk of lung cancer (ρ<0) included high education levels (the proportion of people who received college or associate’s degree or bachelor’s and higher degree), high median household income, high proportion of population within half a mile of a park, and private insurance coverage. The high-risk category had a higher proportion of low-income or low-education populations (the percentage of a combination of the population aged 18-24 years with less than high school graduate diploma education and the population aged ≥25 years with less than 12th grade diploma education in the area) than the low-risk category (Multimedia Appendix 6), indicating a health disparity related to lung cancer.

## Discussion

### Summary of Main Findings

In this study, we developed and prospectively validated a risk prediction model of the future 1-year incidence of lung cancer using EHR data derived from more than 1.1 million people in the state of Maine. Patients were stratified into three risk categories, ranking the lung cancer risk as low, medium, and high. The model achieved an AUC of 0.881 (95% CI 0.873-0.889) in the prospective cohort, indicating our model’s ability to target those most at risk for subsequent prevention management. The incidence of lung cancer in the high-risk category (579/53,922, 1.07%) was 7.7 times higher than that in the overall prospective cohort (1167/836,659, 0.14%). Performance of the model in subgroups (Figure 2), especially those considered low risk by prior models (age<45 years), was fairly good (AUC is 0.880), showing predictive power in patients that traditional models tend to ignore. Key parameters of age, a history of pulmonary diseases and other chronic diseases, medications for mental disorders, and social disparities were found to be significantly associated with incident lung cancer.

### Comparison With Prior Work

We compared our model with seven other risk prediction models for incident lung cancer (Multimedia Appendix 12). The models achieved AUCs between 0.57 and 0.87, but with limitations in targeted clinical application. The Bach model [20] and PLCO_m2012_ (Prostate, Lung, Colorectal, and Ovarian Cancer Screening Trial) model [19] were only applicable to smokers. The Liverpool Lung Project model [12] and two Spitz models [17,21] were developed with case-control matched studies with small sample sizes that were not validated with a general population. The Extended Spitz model [17] required genetic test information, which was unavailable in routine clinical data. The EPIC (European Prospective Investigation into Cancer and Nutrition) [15] and HUNT (Helseundersøkelsen i Nord-Trøndelag) [10] models used smoking status (eg, smoking duration, smoking intensity, and years since cessation) collected from a questionnaire as a predictor, which may not be feasible in a large, general population. Furthermore, our model had a short prediction time frame (1 year) compared with most other studies where the follow-up periods were up to several years. A short prediction time frame resulted in a low incidence (0.12% in the retrospective cohort and 0.14% in prospective cohort), which raised the challenge of prediction. To address this issue, our model adopted more predictors (118 features) than prior risk models (<15 features), making our risk prediction more effective.

### Interpretation of Risk Predictors and Implications for Prevention and Early Intervention

#### Pulmonary Diseases and Inflammation Markers

A total of 27.7% (14,972/53,922) individuals in the high-risk group had one or more pulmonary diseases, which was much higher than the number in the low-risk population (3.39%, 22,832/673,075). The association between the pulmonary diseases and lung cancer was reported in many previous studies [49-52]. Pulmonary diseases induced an inflammatory response in the lung, and inflammation played a critical role in the development of lung cancer [53-56]. The C-reactive protein level and leukocyte count are blood test markers for inflammation. Elevated C-reactive protein levels and leukocytes counts have been found to be associated with pulmonary diseases [57,58] and lung cancer [59], suggesting an etiologic role of pulmonary inflammation in lung cancer pathophysiology [59]. Consistent with these studies, our model recognized abnormal C-reactive protein levels and leukocyte counts as top features (Multimedia Appendix 5). In the high-risk category, 2.52% (1358/53,922) individuals had abnormal C-reactive protein levels and 19.98% (10,774/53,922) had high leukocytes counts compared with 0.7% (4711/673,075) and 6.27% (42,202/673,075) in the low-risk category, respectively (Multimedia Appendix 6).

#### Combination of Pulmonary Diseases and Other Chronic Diseases

Our study showed that more than 80% (43,165/53,922) of individuals in the high-risk category had at least one chronic disease, and the majority (75.41%, 507,565/673,075) of the low-risk population had no chronic disease diagnosis. Moreover, 22.05% (11,890/53,922) of the high-risk patients had pulmonary diseases together with other chronic diseases (ie, CVDs, CKD, and diabetes), and the risk of incident lung cancer increased among these patients (Multimedia Appendix 8). In addition, the concurrent chronic diseases led to an increased burden of clinical utilization and cost (Multimedia Appendix 10). We also found that incident lung cancer was associated with abnormal results of many chronic disease markers (eg, glomerular filtration rate, glucose level, and platelet count) and a group of previously prescribed medications including drugs for pulmonary diseases (Ipratropium bromide, albuterol sulfate, etc), drugs for diabetes (metformin HCl, glipizide, etc), and drugs for CVDs (amlodipine besylate, diltiazem HCl, valsartan, etc). Such markers and medication histories indicated that patients at risk for or living with diseases might develop lung cancer. These findings were consistent with those of previous studies. High chronic disease burden is a growing concern in the US population. It was reported that 6/10 adult Americans have at least one chronic disease and 4/10 have more than one chronic disease [60,61]. A recent study found that chronic diseases are an overlooked risk factor for cancer, and a substantial cancer risk is associated with a combination of cardiovascular disease markers, diabetes, chronic kidney disease markers, and pulmonary diseases [62], which were found to be linked to the risk of the next-year incident lung cancer by our model.

#### Mental Disorders

Mental disorders may affect the immune system and endocrine function, thus influencing the body’s susceptibility to cancer [63]. Several studies showed a positive association between mental disorders and the overall risk of cancer [63,64]. For lung cancer, some studies showed an etiological association [65,66], whereas some claimed there was no association between mental disorders and lung cancer [67,68]. We explored the role of this controversial and unclear association in our study.

Due to the data policy of the EHR data on mental illness in the state of Maine, we used the consumption of mental illness–related drugs as a proxy for mental disorders. We found that mental disorders had a positive association with the 1-year lung cancer incidence risk (Multimedia Appendix 9). Patients with no chronic diseases who were undergoing treatment for mental disorders had nearly 2.5 times the risk of incident lung cancer compared to those without any mental disorders, regardless of age, gender, and smoking status. This can be explained by a previous finding that adverse psychological events such as pressure and stress may impair the immune system and cause the development and progression of tumors [52]. A similar correlation was also observed in a study focusing on behavioral immunological activities: Researchers found that unpleasant or hostile emotions could cause immune system disorders, and consequently, the occurrence of tumors [53]. More interestingly, those psychological events were found to be correlated to smoking: People with a diagnosis of posttraumatic stress disorder were found to have a higher rate of smoking (45%) than people without a mental health diagnosis (23%) [69]. Smoking is an important factor of lung cancer. Therefore, close attention should be paid to those with a high-risk mental status to allow prevention of and intervention for lung cancer.

#### Social Determinants

A few studies have shown that lung cancer risk is inversely associated with socioeconomic status factors such as educational attainment, income, and occupation [70]. Socioeconomic status was found to be linked with health status through multiple pathways such as social resources, physical and psychosocial stressors, and health-related behaviors [71]. Consistent with these studies, our model found that patients with low income or less education had a higher risk of lung cancer (Multimedia Appendix 11). Living distance to parks and coverage by Medicaid were also risk factors in our model. The former may be explained by the fact that physical environment factors such as the concentration of parks in the living area can directly shape peoples’ physical activities and ultimately decrease the lung cancer risk. Low education level could be the causality for future low family income, less access to health care, and attainment of Medicaid health insurance.

### Implications of Findings

The predictive model and risk scores can benefit health care organizations at multiple levels. For health care providers, stratifying the population by our risk score will help with budget planning and target intervention. For clinicians, the model can be used as an assistant tool for decision making. Our model can also act as a prescreening tool: High-risk patients identified by our model can be referred to the LDCT screening test to decide whether the patients already have lung cancer.

The ultimate goal of this study is to guide health care providers to make decisions for the prevention and intervention of lung cancer. There are already established guidelines in lung cancer preventive care to address both nonmodifiable and modifiable risk factors. The modifiable risk factors such as concurrent chronic conditions and lifestyles are even more important than nonmodifiable predictors such as age and gender, as they offer an opportunity to both clinicians and patients to proactively manage the disease by implementing interventions before deterioration.

Our model identified 68.02% (470/691) of high-risk patients at least 3 months before the confirmatory diagnosis was made by physicians. This may provide the opportunity of early interventions to prevent or delay the development of lung cancer as well as to reduce corresponding health care expenditures. Early detection of lung cancer can lead to an improved 5-year survival rate [3]. In addition, a recent study revealed that patients who received anti-inflammatory therapy had a marked reduction in the incidence of lung cancer [72]. Another route of intervention is through the management of multiple chronic diseases. Recent studies showed a substantial impact of chronic diseases (eg, cardiovascular diseases, diabetes, chronic kidney diseases, and pulmonary diseases) jointly on cancer risk, which was as important as five lifestyle factors combined (smoking, unhealthy diet, physical inactivity, obesity, and alcohol misuse) [62]. Better management of chronic diseases in primary care is therefore an effective strategy for future cancer prevention. In addition, increasing physical activity is a way to improve lifestyle. Our model found that patients living far away from a park were prone to an elevated risk of lung cancer, indicating that targeting this subgroup of patients with personalized action plans might lead to healthier life styles and a possible reduced risk of lung cancer.

### Limitations

Our study has several limitations. First, some data were missing in our dataset. Tobacco use was not fully recorded in the EHR data; occupational exposure and family history of lung cancer were so sparse in the data source that our model did not include them as predictors; and patients with lung cancer might not have any record of this diagnosis, leading to an underestimation of lung cancer prevalence in the study. Second, air quality, cancer biomarker, and some individual-level lifestyle information (eg, diet habit and physical activity) could be potentially useful predictors for development of lung cancer, but in EHRs, these data were not available. Third, the grade and stage of lung cancer were not described in the data source, and the socioeconomic factors were analyzed at a community-level, limiting the findings between the association of the individual socioeconomic status and lung cancer.

### Conclusions

A risk prediction model of the future 1-year incidence of lung cancer was developed and prospectively validated using the preceding 6 months’ EHR data derived from more than 1.1 million people in the state of Maine. The model was able to assign each individual a risk score and stratified patients into three risk categories of low, medium, and high risk. The model reached an AUC of 0.881 in the prospective cohort. Age, a history of pulmonary diseases and other chronic diseases, medications for mental disorders, and social disparities were found to be associated with new incident lung cancer. Targeting individuals at high risk has the potential to facilitate early intervention and reduce overall costs, which will ultimately benefit patients, clinicians, and health care providers.

## Appendix

Multimedia Appendix 1 List of social determinant variables downloaded from the US census, with details of the data source and mapping. method.

Multimedia Appendix 2 Comparative analysis of the model performance, quantified by the area under the curve (AUC) with 95% CI. (1) Our Extreme Gradient Boosting (XGBoost) algorithm, (2) RandomForest, (3) Boosting, (4) Support Vector Machine (SVM), (5) LASSO, and (6) K-Nearest Neighbors (KNN).

Multimedia Appendix 3 Comparison of the predictive performances to predict future 1-year risk of new incident lung cancer in the prospective cohort, measured by the receiver operating characteristic area under the curve (ROC AUC): Model 1 is based on our feature selection method, Model 2 is with the Gini index, and Model 3 is with information gain feature selection methods.

Multimedia Appendix 4 The performance of the 1-year lung cancer risk prediction model in the prospective cohort, summarized as positive prediction value, sensitivity, specificity, and mean relative risk.

Multimedia Appendix 5 The top 60 features selected by our lung cancer prediction model.

Multimedia Appendix 6 Distribution of top risk predictors across the three risk categories.

Multimedia Appendix 7 Constituent ratios of age subgroups across the identified three risk categories.

Multimedia Appendix 8 The time-to-diagnosis curves for patients who only had pulmonary diseases and patients who had pulmonary diseases together with at least one other chronic disease in the prospective cohort.

Multimedia Appendix 9 Predicted time-to-diagnosis curves for patients who had at least one diagnosis of chronic diseases and those who did not receive a diagnosis of chronic diseases. Curves for both subgroups were stratified by mental health status (Mental Disorder vs No Mental Disorder).

Multimedia Appendix 10 Graph of patients’ average clinical costs in the past 6 months against the average number of chronic diseases.

Multimedia Appendix 11 Spearman rank correlation between six social determination features and prospective lung cancer risk scores.

Multimedia Appendix 12 Characteristics of the compared risk models.

## Abbreviations

AUC: area under the curve
CKD: chronic kidney disease
COPD: chronic obstructive pulmonary disease
CVD: cardiovascular disease
EHR: electronic health record
EPIC: European Prospective Investigation into Cancer and Nutrition
HIE: health information exchange
HR: hazard ratio
HUNT: Helseundersøkelsen i Nord-Trøndelag
ICD-10-CM: International Classification of Diseases, 10th Revision, Clinical Modification
LDCT: low-dose computed tomography
OR: odds ratio
PLCO_m2012_: Prostate, Lung, Colorectal, and Ovarian Cancer Screening Trial
PPV: positive predictive value
