# Supplementary material for: Prediction of the 1-Year Risk of Incident Lung Cancer: Prospective Study Using Electronic Health Records from the State of Maine
Source: J Med Internet Res. 2019 May 16;21(5):e13260. doi: 10.2196/13260 (PMC6542253; doi:10.2196/13260)
Supplement: Multimedia Appendix 1 [file jmir_v21i5e13260_app1.pdf]

Multimedia Appendix 1

List of social determinant variables downloaded from the US census, detailed in the data source and mapping method.

	Social Determinants	Levels	Variable Type	Mapping Method	Data Source
1	Racial Demographics	Four levels: White; Black/African American; American Indian; Asian	%	ZIP code	US Census ACS (American Community Survey)
2	Urban or Rural	Two levels: Urban ratio; Rural ratio	%	ZIP code	
3	Nativity and Citizenship		%	ZIP code	
4	Education level	Four levels: Less than High school; High School; College associate bachelor; Graduate degree.	%	ZIP code	
5	English proficiency		%	ZIP code	
6	Health insurance status	Six levels:<br>Public: Medicare; Medicaid; VA health care.<br>Private: Employ based; Direct purchase; Tricare/military.	%	ZIP code	
7	Poverty or Household income		%	ZIP code	
8	Unemployment rate		%	ZIP code	
9	Percentage of population living within half mile to park		%	County level	Centers for Disease Control and Prevention, Community Health Status Indicators (CDC CHSI)
10	GINI inequality index		[0,1]	ZIP code	US Census (ACS)
11	Social Vulnerability Index		[0,1]	County level	CDC or Agency for Toxic Substances and Disease Registry (ATSDR)
