# Supplementary material for: Prediction of the 1-Year Risk of Incident Lung Cancer: Prospective Study Using Electronic Health Records from the State of Maine
Source: J Med Internet Res. 2019 May 16;21(5):e13260. doi: 10.2196/13260 (PMC6542253; doi:10.2196/13260)

Multimedia Appendix 2

Comparative analysis of the model performance, quantified by the AUC with 95% CI: 1. Our XGBoost algorithm; 2. RandomForest; 3. Boosting; 4. Support Vector Machine (SVM); 5. LASSO; and 6. K-Nearest Neighbors (KNN).

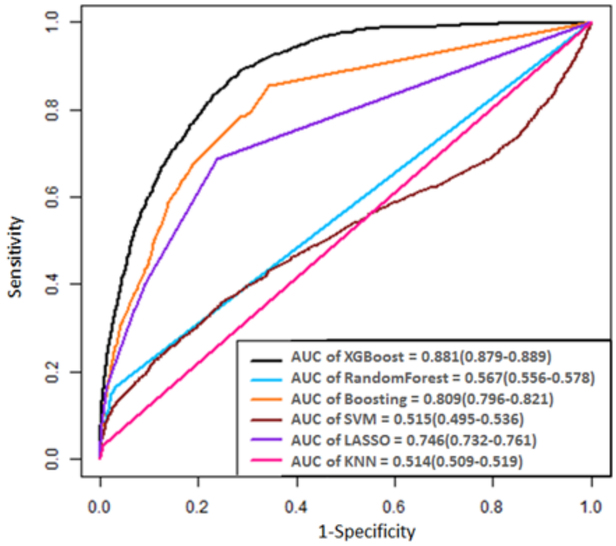

Supplement: Multimedia Appendix 2 [file jmir_v21i5e13260_app2.pdf]
