# Supplementary material for: Prediction of the 1-Year Risk of Incident Lung Cancer: Prospective Study Using Electronic Health Records from the State of Maine
Source: J Med Internet Res. 2019 May 16;21(5):e13260. doi: 10.2196/13260 (PMC6542253; doi:10.2196/13260)

Multimedia Appendix 3

Predictive performances to predict future one-year risk of new incident lung cancer in the prospective cohort, measured by the ROC AUC, were compared: Model 1 - based on our feature selection method; Model 2 - with gini index; Model 3 - with information gain feature selection methods.

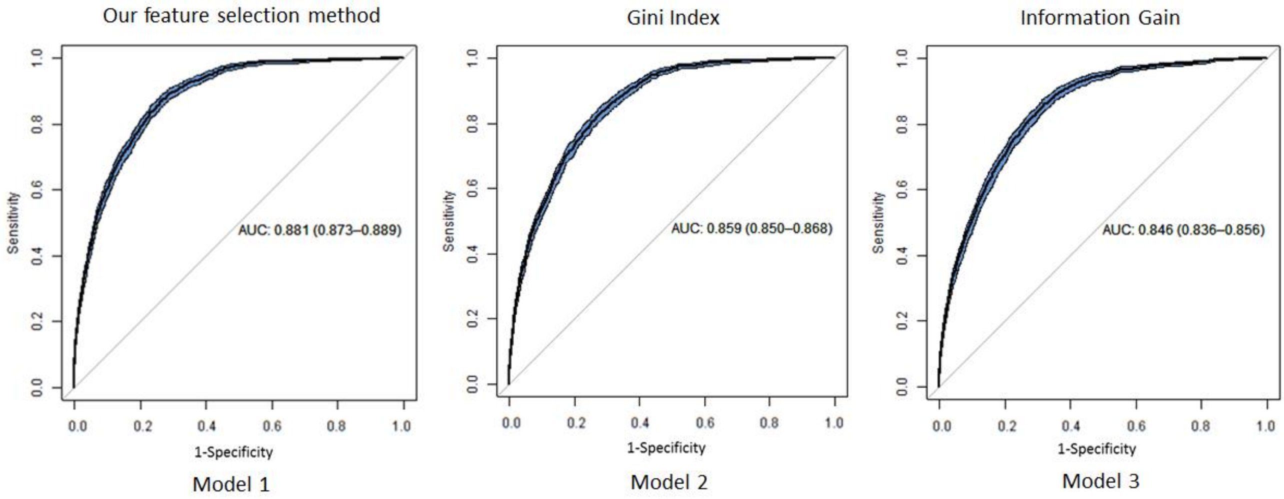

Supplement: Multimedia Appendix 3 [file jmir_v21i5e13260_app3.pdf]
