# Supplementary material for: Prediction of the 1-Year Risk of Incident Lung Cancer: Prospective Study Using Electronic Health Records from the State of Maine
Source: J Med Internet Res. 2019 May 16;21(5):e13260. doi: 10.2196/13260 (PMC6542253; doi:10.2196/13260)
Supplement: Multimedia Appendix 4 [file jmir_v21i5e13260_app4.pdf]

Multimedia Appendix 4

The performance of the one-year lung cancer risk prediction model in the prospective cohort, summarized in PPV, sensitivity, specificity and mean relative risk.

**Risk Category**	**Low**	**Medium**	**High**	**Total**
**Intervals**	[0-0.0045]	[0.0045-0.01]	[0.01-1]	
**Number of Patient**	673,075	109,662	53,922	836,659
**True Positive**	262	326	579	1,167
**PPV (%)**	0.04	0.3	1.07	
**Sensitivity (%)**	22.45	27.93	49.61	
**Specificity(%)**	80.53	13.09	6.38	
**Mean Relative Risk**	0.28	2.13	7.7	
