# Supplementary material for: Prediction of the 1-Year Risk of Incident Lung Cancer: Prospective Study Using Electronic Health Records from the State of Maine
Source: J Med Internet Res. 2019 May 16;21(5):e13260. doi: 10.2196/13260 (PMC6542253; doi:10.2196/13260)
Supplement: Multimedia Appendix 5 [file jmir_v21i5e13260_app5.pdf]

Multimedia Appendix 5

The top 60 features selected by our lung cancer prediction model.

Characteristic	Prospective cohort (N=836,659)	Case (N=1,167) n (%)	Control (N=835,492) n (%)	OR(95% CI)	P value
**Age**					
<45	366,752	20 (1.71%)	366,732 (43.89%)	0.022(0.014-0.034)	<0.001
45-54	109,986	70 (6.00%)	109,916 (13.16%)	0.369(0.278-0.480)	<0.001
55-64	139,219	241 (20.65%)	138,978 (16.63%)	1.206(1.040-1.392)	0.012
⩾65	220,702	836 (71.64%)	219,866 (26.32%)	7.480(6.567-8.544)	<0.001
**Gender**					
Male	369,022	558 (47.81%)	368,464 (44.10%)	1.16(1.04-1.30)	0.011
**Smoking**	14,248	65 (5.57%)	14,183 (1.70%)	4.084(3.144-5.21)	<0.001
**Pulmonary Diseases**					
COPD	36,221	354 (30.33%)	35,867 (4.29%)	4.978 (4.379-5.648)	<0.001
Pneumonia	12,179	71 (6.08%)	12,108 (1.45%)	2.79 (2.172-3.525)	<0.001
Other respiratory disorders	5,743	65 (5.57%)	5,678 (0.68%)	5.484 (4.219-7)	<0.001
**Other cancer history**	72,073	339 (29.05%)	71,734 (8.59%)	1.899 (1.667-2.159)	<0.001
**Other chronic diseases**					
Diabetes	70,005	230 (19.71%)	69,775 (8.35%)	1.329 (1.146-1.535)	<0.001
CVDs	161,685	524 (44.90%)	161,161 (19.29%)	1.374 (1.219-1.548)	<0.001
CKD	18,912	81 (6.94%)	18,831 (2.25%)	1.27 (1.004-1.585)	0.040
**Symptoms**					
Haemoptysis	982	10 (0.86%)	972 (0.12%)	5.08 (2.528-9.001)	<0.001
Pleural effusion	2,356	29 (2.49%)	2,327 (0.28%)	4.13 (2.783-5.874)	<0.001
Cough	36,991	112 (9.60%)	36,879 (4.41%)	2.108 (1.724-2.55)	<0.001
Abnormal weightloss	5,804	24 (2.06%)	5,780 (0.69%)	2.01 (1.303-2.944)	0.001
Dyspnea	3,757	18 (1.54%)	3,739 (0.45%)	1.877 (1.133-2.901)	0.008
Chest pain	4,396	15 (1.29%)	4,381 (0.52%)	1.523 (1.217-1.882)	<0.001
**Abnormal laboratory tests**					
C reactive protein	8,520	27 (2.31%)	8,493 (1.02%)	1.771 (1.178,2.541)	0.003
Leukocytes count	71,748	185 (15.85%)	71,563 (8.57%)	1.483 (1.263,1.732)	<0.001
Platelets	51,499	145 (12.43%)	51,354 (6.15%)	1.496 (1.251,1.775)	<0.001
Glomerular filtration rate	18,711	62 (5.31%)	18,649 (2.23%)	1.387 (1.062,1.776)	0.013
Glucose	103,764	318 (27.25%)	103,446 (12.38%)	1.623 (1.423,1.846)	<0.001
Neutrophils	60,292	177 (15.17%)	60,115 (7.20%)	1.705 (1.447,1.996)	<0.001
Monocytes	53,911	159 (13.62%)	53,752 (6.43%)	1.63 (1.373,1.922)	<0.001
Lymphocytes	52,227	159 (13.62%)	52,068 (6.23%)	1.611 (1.357,1.9)	<0.001
Eosinophils	46,278	122 (10.45%)	46,156 (5.52%)	1.501 (1.238,1.804)	<0.001
Alkaline phosphatase	56,915	161 (13.80%)	56,754 (6.79%)	1.517 (1.279,1.787)	<0.001
**Medication of pulmonary diseases**					
Albuterol sulfate	5,084	16 (1.37%)	5,068 (0.61%)	5.708 (3.324,9.069)	<0.001
Ipratropium bromide	1,402	7 (0.60%)	1,395 (0.17%)	3.093 (1.323,6.027)	0.003
Ciprofloxacin hcl	10,626	39 (3.34%)	10,587 (1.27%)	1.816 (1.296,2.466)	<0.001
Levofloxacin	7,425	67 (5.74%)	7,358 (0.88%)	3.859 (2.982,4.907)	<0.001
**Medicine of diabetes**					
Metformin hcl	21,892	91 (7.80%)	21,801 (2.61%)	1.933 (1.549,2.382)	<0.001
Glipizide	10,993	47 (4.03%)	10,946 (1.31%)	1.51 (1.111,2)	0.006
**Medication of CVDs**					
Amlodipine besylate	19,490	118 (10.11%)	19,372 (2.32%)	2.231 (1.832,2.691)	<0.001
Diltiazem hcl	1,990	14 (1.20%)	1,976 (0.24%)	2.248 (1.26,3.665)	0.003
Valsartan	3,953	23 (1.97%)	3,930 (0.47%)	1.933 (1.24,2.853)	0.002
Losartan potassium	13,337	64 (5.48%)	13,273 (1.59%)	1.74 (1.337,2.222)	<0.001
Metoprolol tartrate	18,617	93 (7.97%)	18,524 (2.22%)	1.792 (1.438,2.205)	<0.001
Lisinopril	48,878	215 (18.42%)	48,663 (5.82%)	1.889 (1.623,2.189)	<0.001
Hydrochlorothiazide	17,112	84 (7.20%)	17,028 (2.04%)	2.054 (1.631,2.551)	<0.001
Atenolol	13,394	68 (5.83%)	13,326 (1.59%)	1.804 (1.397,2.289)	<0.001
**Medicine of mental disorders**					
Trazodone	16,122	71 (6.08%)	16,051 (1.92%)	2.611 (2.033,3.297)	<0.001
Sertraline	23,453	83 (7.11%)	23,370 (2.80%)	2.148 (1.704,2.671)	<0.001
Mirtazapine	6,077	25 (2.14%)	6,052 (0.72%)	1.997 (1.306,2.905)	0.001
Lorazepam	16,397	84 (7.20%)	16,313 (1.95%)	2.825 (2.242,3.509)	<0.001
Diazepam	7,487	25 (2.14%)	7,462 (0.89%)	2.177 (1.424,3.165)	<0.001
Bupropion	14,384	42 (3.60%)	5,654 (0.68%)	2.27 (1.641,3.051)	<0.001
Aripiprazole	3,629	9 (0.77%)	3,620 (0.43%)	2.745 (1.313,4.981)	0.003
Alprazolam	8,228	26 (2.23%)	8,202 (0.98%)	1.686 (1.112,2.436)	0.009

Characteristic	Prospective cohort (N=836,659)	Case (N=1,167)	Control (N=835,492)	Coefficients(95% CI)	P value
**Utilizations in the last 6 months**					
Patient's estimated cost (US $)	1629.599514	4330.238218	1625.827311	2128.869 (1756.116,2501.622)	<0.001
Number of chronic conditions	2.81	6.40	2.80	1.573 (1.37,1.775)	<0.001
Mean of outpatient visits per person	3.69	6.11	3.69	1.486 (1.229,1.742)	<0.001
Mean of inpatient days per person	0.40	1.62	0.40	1.022 (0.727,1.316)	<0.001
Mean of inpatient admissions per person	0.08	0.21	0.08	0.104 (0.084,0.125)	<0.001
Mean of emergency visits per person	0.31	0.41	0.31	0.155 (0.107,0.203)	<0.001
**Social Determinants**					
Low-educated population(%)	8.45	8.95	8.45	0.005 (0.003,0.007)	<0.001
High-educated population(%)	9.96	9.36	9.96	-0.01 (-0.013,-0.006)	<0.001
Median household income, ZIP code	49243.84	47387.79	49246.43	-2441.741 (-3320.51,-1562.972)	<0.001
Medicaid coverage(%)	21.13	22.49	21.12	1.438 (0.918,1.957)	<0.001
Private Insurance coverage(%)	52.18	51.19	52.18	-1.148 (-1.778,-0.519)	<0.001
Population within half-mile of park (%)	10.96	10.29	10.96	-0.409 (-0.8,-0.018)	0.040
