# Supplementary material for: Prediction of the 1-Year Risk of Incident Lung Cancer: Prospective Study Using Electronic Health Records from the State of Maine
Source: J Med Internet Res. 2019 May 16;21(5):e13260. doi: 10.2196/13260 (PMC6542253; doi:10.2196/13260)
Supplement: Multimedia Appendix 6 [file jmir_v21i5e13260_app6.pdf]

Multimedia Appendix 6

Distribution of impactful risk predictors across the three risk categories.

Risk category	Low	Medium	High
**Risk score intervals**	[0-0.0045]	[0.0045-0.01]	[0.01-1]
**Population**	673,075	109,662	53,922
**Age**			
<45	54.16%	1.62%	0.79%
45-54	14.96%	6.43%	4.17%
55-64	17.24%	15.38%	11.72%
⩾65	13.64%	76.56%	83.32%
**Gender**			
Male	44.05%	43.77%	45.53%
Female	55.95%	56.23%	54.47%
**Smoking**	1.38%	2.10%	4.96%
**Pulmonary Diseases**			
COPD	1.98%	10.25%	21.62%
Pneumonia	0.94%	2.87%	5.07%
Other respiratory disorders	0.43%	1.36%	2.53%
**Other cancer history**	4.91%	22.39%	26.85%
**Other chronic diseases**			
Diabetes	5.04%	20.95%	24.29%
CVDs	12.30%	47.15%	50.48%
CKD	0.81%	7.23%	10.31%
**Symptoms**			
Haemoptysis	0.08%	0.21%	0.42%
Pleural effusion	0.08%	0.78%	1.74%
Cough	3.78%	5.93%	9.38%
Abnormal weightloss	0.48%	1.40%	1.88%
Dyspnea	0.25%	1.10%	1.55%
Chest pain	3.49%	7.05%	7.18%
**Abnormal laboratory tests**			
C reactive protein	0.70%	2.22%	2.52%
Leukocytes count	6.27%	17.14%	19.98%
Glomerular filtration rate	1.29%	5.57%	7.31%
Glucose	8.52%	27.23%	30.65%
Platelets	4.20%	13.22%	16.15%
Neutrophils	2.42%	7.73%	10.03%
Monocytes	4.46%	13.40%	17.07%
Lymphocytes	4.19%	13.51%	17.09%
Eosinophils	3.98%	10.96%	13.83%
Alkaline phosphatase	4.77%	14.30%	16.96%
**Medication of pulmonary diseases**			
Albuterol sulfate	0.65%	0.35%	0.63%
Ipratropium bromide	0.12%	0.30%	0.52%
Ciprofloxacin hcl	0.84%	2.85%	3.37%
Levofloxacin	0.45%	2.15%	3.82%
**Medicine of diabetes**			
Metformin hcl	1.65%	6.34%	7.14%
Glipizide	0.62%	3.81%	4.89%
**Medication of cardiovascular diseases**			
Amlodipine besylate	1.10%	6.72%	8.69%
Diltiazem hcl	0.08%	0.73%	1.20%
Valsartan	0.23%	1.39%	1.66%
Losartan potassium	0.78%	4.57%	5.73%
Metoprolol tartrate	0.96%	6.45%	9.36%
Lisinopril	3.42%	15.00%	17.50%
Hydrochlorothiazide	1.26%	5.28%	5.29%
Atenolol	0.85%	4.53%	5.06%

Risk category	Low	Medium	High
**Risk score intervals**	[0-0.0045]	[0.0045-0.01]	[0.01-1]
**Population**	673,075	109,662	53,922
**Medicine of mental disorders**			
Trazodone	1.38%	3.88%	4.74%
Sertraline	2.16%	4.94%	6.50%
Mirtazapine	0.45%	1.62%	2.36%
Lorazepam	1.34%	4.18%	5.17%
Diazepam	0.70%	1.65%	1.76%
Bupropion	1.48%	2.65%	2.77%
Aripiprazole	0.39%	0.60%	0.61%
Alprazolam	0.69%	2.06%	2.45%
**Utilizations in the last 6 months**			
Mean of estimated cost per person (US $)	1,209.75	3,275.78	3,522.51
Mean of chronic condition counts per person	1.80	6.42	8.07
Mean of outpatient visits per person	3.06	6.18	6.51
Mean of inpatient days per person	0.25	0.95	1.12
Mean of inpatient admissions per person	0.05	0.17	0.19
Mean of emergency visits per person	0.28	0.38	0.42
**Social Determinants**			
Low-educated population(%)	8.64	8.96	9.23
High-educated population(%)	49.10	48.20	47.88
Median household income, ZIP code	51,646.07	49,019.50	47,417.20
Medicaid coverage(%)	21.57	22.79	23.68
Private Insurance coverage(%)	54.32	52.19	51.76
Population within half-mile of park (%)	11.52	10.59	10.17
