# Supplementary material for: Prediction of the 1-Year Risk of Incident Lung Cancer: Prospective Study Using Electronic Health Records from the State of Maine
Source: J Med Internet Res. 2019 May 16;21(5):e13260. doi: 10.2196/13260 (PMC6542253; doi:10.2196/13260)

Multimedia Appendix 7

Constituent ratios of age subgroups across the identified three risk categories.

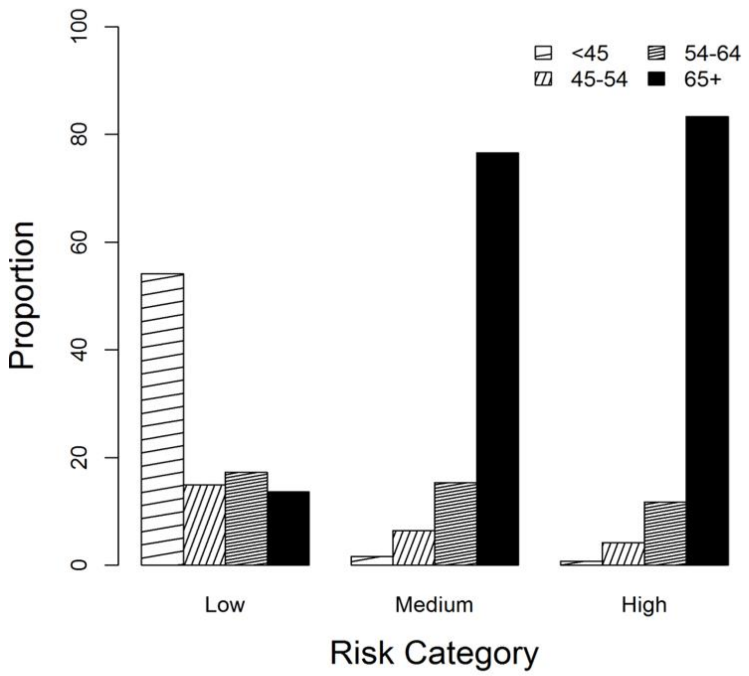

Supplement: Multimedia Appendix 7 [file jmir_v21i5e13260_app7.pdf]
