# Supplementary material for: Prediction of the 1-Year Risk of Incident Lung Cancer: Prospective Study Using Electronic Health Records from the State of Maine
Source: J Med Internet Res. 2019 May 16;21(5):e13260. doi: 10.2196/13260 (PMC6542253; doi:10.2196/13260)

Multimedia Appendix 8

The time-to-diagnosis curves for patients who only had pulmonary diseases and patients who had pulmonary diseases together with at least one other chronic disease in the prospective cohort.

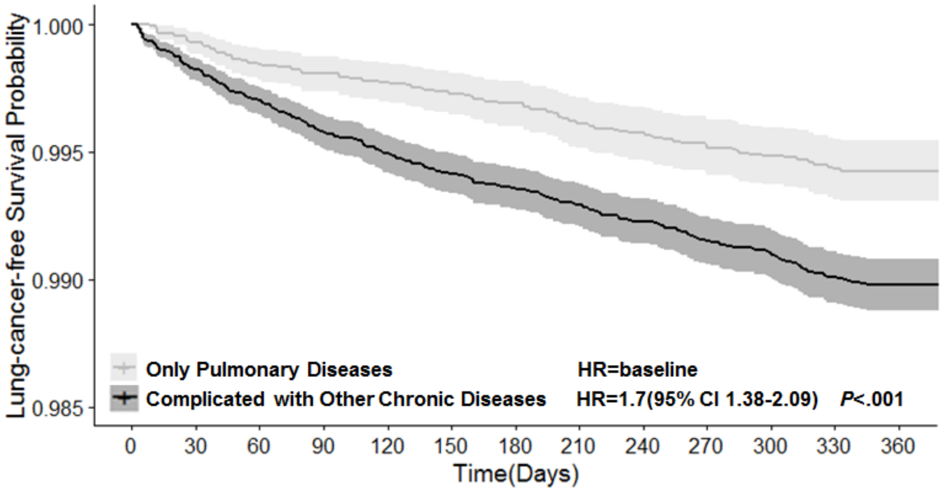

Supplement: Multimedia Appendix 8 [file jmir_v21i5e13260_app8.pdf]
