# Supplementary material for: Prediction of the 1-Year Risk of Incident Lung Cancer: Prospective Study Using Electronic Health Records from the State of Maine
Source: J Med Internet Res. 2019 May 16;21(5):e13260. doi: 10.2196/13260 (PMC6542253; doi:10.2196/13260)

Multimedia Appendix 9

Predicted time-to-diagnosis curves for patients who had at least one diagnosis of chronic diseases and who received no diagnosis of chronic diseases. Curves for both subgroups were stratified by mental health status (Mental Disorder vs No Mental Disorder). [a]adjusted for age, gender and smoking.

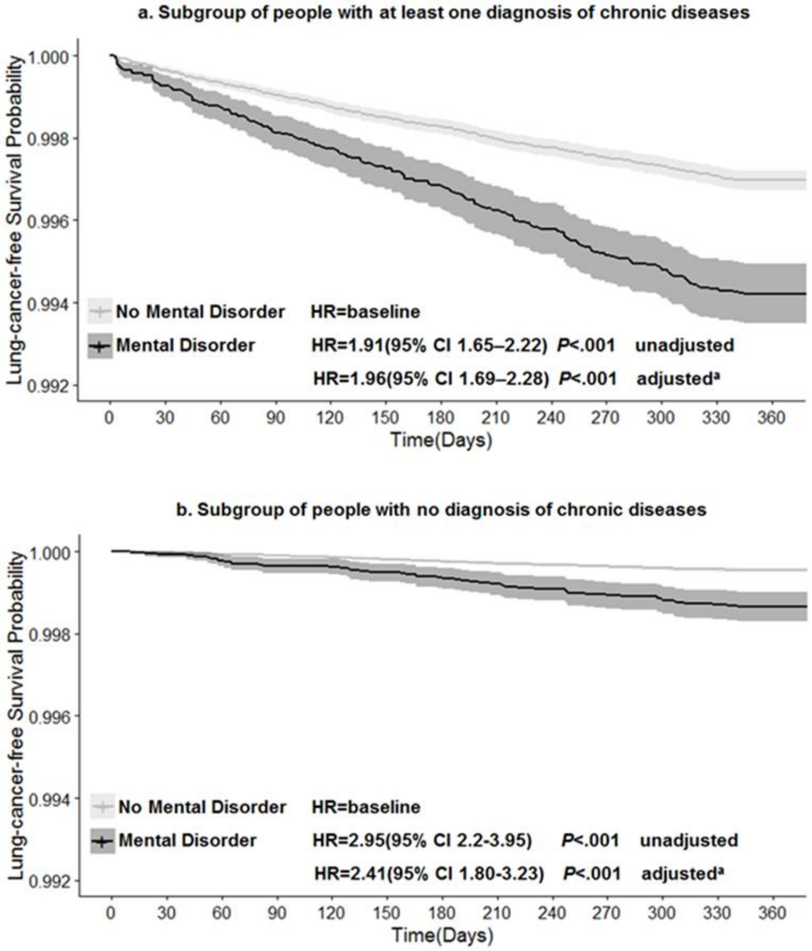

Supplement: Multimedia Appendix 9 [file jmir_v21i5e13260_app9.pdf]
