# Supplementary material for: Prediction of the 1-Year Risk of Incident Lung Cancer: Prospective Study Using Electronic Health Records from the State of Maine
Source: J Med Internet Res. 2019 May 16;21(5):e13260. doi: 10.2196/13260 (PMC6542253; doi:10.2196/13260)

Multimedia Appendix 10

Patients' average clinical costs in the past 6 months against the average number of chronic diseases. The circles were formed by 8 common disease subgroups under the low-risk (green circle) and high-risk (red circle) categories, respectively. The circle size indicates the proportion of the disease subgroup under each risk category. The 8 chronic diseases were COPD, CVDs, pneumonia, other respiratory disorders, diabetes, CKD, mental disorders, and other cancer history. Reference groups consisted of patients with no diagnosis of any of the above chronic diseases.

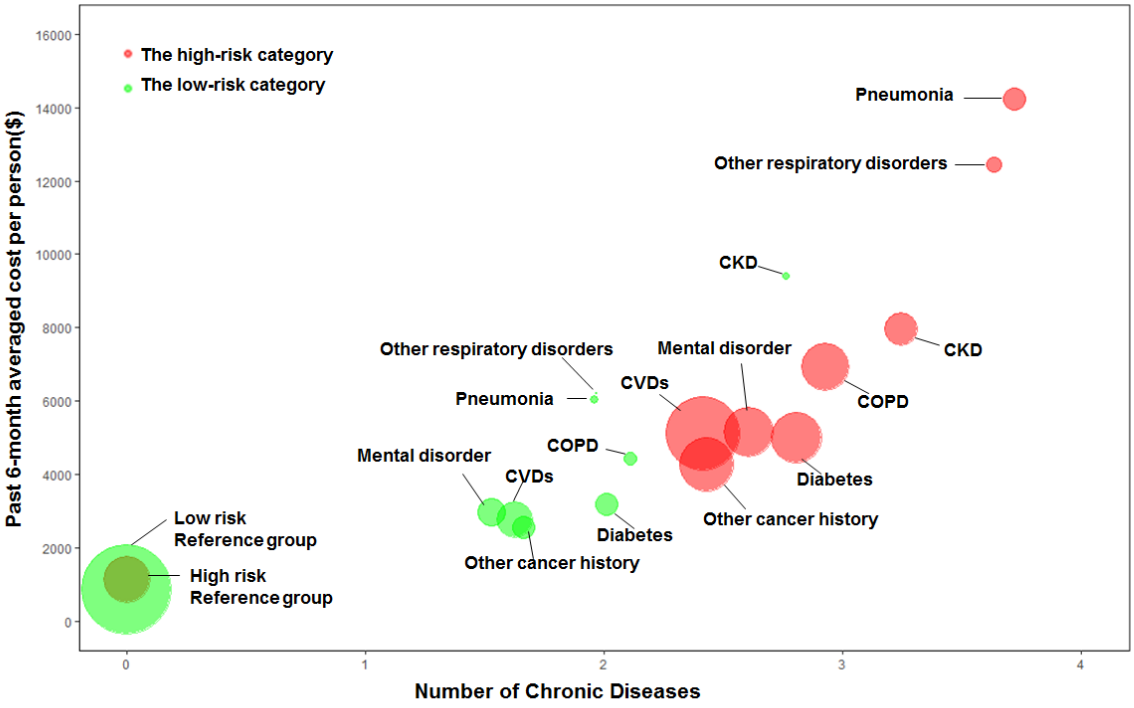

Supplement: Multimedia Appendix 10 [file jmir_v21i5e13260_app10.pdf]
