# Supplementary material for: Prediction of the 1-Year Risk of Incident Lung Cancer: Prospective Study Using Electronic Health Records from the State of Maine
Source: J Med Internet Res. 2019 May 16;21(5):e13260. doi: 10.2196/13260 (PMC6542253; doi:10.2196/13260)

Multimedia Appendix 11

Spearman rank correlation between 6 social determination features and prospective lung cancer risk scores. Low-educated population: the percentage of the a combination of the 18-24 year old population with less than high school graduate diploma and ≥25-year-old population with less than 12th grade diploma in the area; High-educated population: the proportion of people who received college or associate's degree or bachelor's and higher degree.

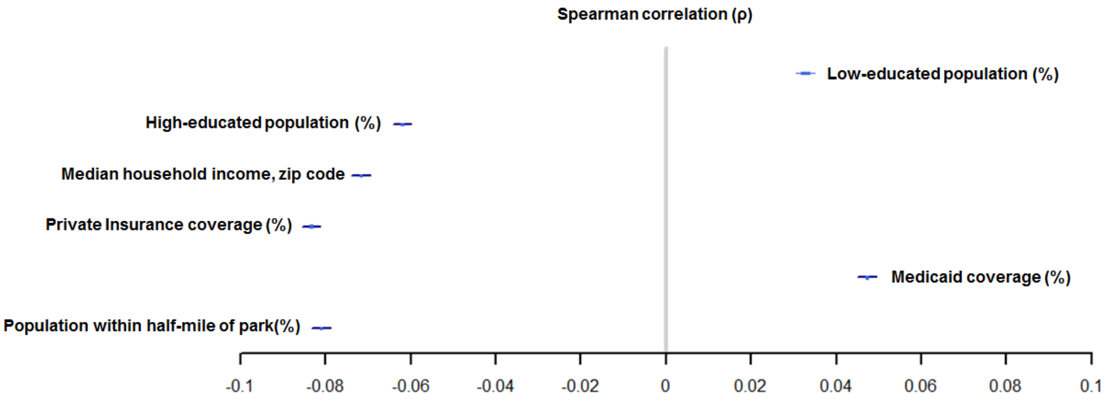

Supplement: Multimedia Appendix 11 [file jmir_v21i5e13260_app11.pdf]
