# Supplementary material for: Prediction of the 1-Year Risk of Incident Lung Cancer: Prospective Study Using Electronic Health Records from the State of Maine
Source: J Med Internet Res. 2019 May 16;21(5):e13260. doi: 10.2196/13260 (PMC6542253; doi:10.2196/13260)
Supplement: Multimedia Appendix 12 [file jmir_v21i5e13260_app12.pdf]

**Multimedia Appendix 12**

Characteristics of compared risk models. NR means not reported.

| Model | Reference | Prediction time frame | Dataset and Size(N) | Risk factors | Eligibility Criteria | AUC | Sensitivity | Specificity |
|---|---|---|---|---|---|---|---|---|
| Bach Model | Bach et al. 2003 [20] | 10-year | Carotene and Retinol Efficacy Trial (CARET), N=18,172 | Age, gender, smoking duration, smoking intensity, years since cessation, asbestos exposure | Aged 45-69 years, ever smokers, 20 pack-year history, quit within 15 years | 0.72 | NR | NR |
| LLP model | Cassidy et al. 2008 [12] | 5-year | Liverpool Lung Project (LLP) case control study, N=1736 | Age, gender, smoking duration, personal history of cancer, family history of lung cancer, personal history of pneumonia, asbestos exposure | Aged 20-80 years | 0.71 | 0.62 | 0.70 |
| Spitz and Extended Spitz Model | Spitz et al. 2007 [21] and Spitz et al. 2008 [17] | 1-year | Thoracic Center at The University of Texas M. D. Anderson Cancer Center, N=3852(Spitz Model) /1340(Extended Spitz Model) | Age, gender, smoking history, asbestos exposure, exposure to second-hand smoke, medical history, family history of cancer; DNA capacity and bleomycin sensitivity (Extended Spitz Model only) | None | 0.57-0.7 | NR | NR |

| | | | | | | | | |
|---|---|---|---|---|---|---|---|---|
| PLCO $_{m2012}$ model | Tammemagi et al. 2013 [19] | 6-year | Prostate, Lung, Colorectal and Ovarian Cancer Screening Trial (PLCO), N=80,375 | Age, race, education, BMI, COPD, personal history of cancer, family history of lung cancer, smoking status, smoking duration, smoking intensity, years since cessation | Ever smokers, 30 pack-year history | 0.797 | 0.830 | 0.629 |
| EPIC Model | Hoggart et al. 2012 [15] | 1-year and 5-year | European Prospective Investigation into Cancer and Nutrition (EPIC), N= 169,035 | Age started smoking, smoking duration, smoking intensity, 10 occupational/environmental exposures, single-nucleotide polymorphisms | None | 0.845 | NR | NR |
| HUNT Model | Markaki et al. 2018 [10] | 6-year | HUNT2 population in Norway, N=45,341 | age, pack-years, smoking intensity, years since smoking cessation, body mass index, daily cough, and hours of daily indoors exposure to smoke | None | 0.87 | 0.8185 | 0.7831 |
| Our model | | 1-year | Maine Health Information Exchange, N=836,659 | 118 EHR features during the preceding 6 months | None | 0.881 | 0.8363 | 0.7681 |
